# Prevalence, awareness, treatment, and control of dyslipidemia among diabetes mellitus patients and predictors of optimal dyslipidemia control: results from the Korea National Health and Nutrition Examination Survey

**DOI:** 10.1186/s12944-021-01455-3

**Published:** 2021-03-26

**Authors:** Seung Jae Kim, Oh. Deog Kwon, Kyung-Soo Kim

**Affiliations:** 1 Department of Family Medicine, Seoul St. Mary’s Hospital, College of Medicine, The Catholic University of Korea, Seoul, Republic of Korea; 2 International Healthcare Center, Seoul St. Mary’s Hospital, College of Medicine, The Catholic University of Korea, Seoul, Republic of Korea; 3 Republic of Korea Navy 2nd Fleet Medical Corps, Pyeongtaek-si, Gyeonggi-do Republic of Korea

**Keywords:** Diabetes mellitus, Dyslipidemia, Prevalence, Awareness, Treatment, Optimal control, Predictors

## Abstract

**Background:**

This study aimed to investigate the prevalence, awareness, treatment, and control rates of dyslipidemia and identify the predictors of optimal control (low-density lipoprotein cholesterol < 100 mg/dL) among patients with diabetes mellitus (DM).

**Methods:**

A cross-sectional study was conducted using the representative Korea National Health and Nutrition Examination Survey (2014–2018). Overall, 4311 patients with DM, aged ≥19 years, and without cardiovascular diseases were selected, and the prevalence, awareness, treatment, and control rates of dyslipidemia were calculated. Univariate and multivariate logistic regression analyses were conducted to evaluate the factors influencing the optimal control of dyslipidemia.

**Results:**

Dyslipidemia was prevalent in 83.3% of patients with DM, but the awareness and treatment rates were 36.5 and 26.9%, respectively. The control rate among all patients with dyslipidemia was 18.8%, whereas it was 61.1% among those being treated. Prevalence and awareness rates were also significantly higher in women than in men. Dyslipidemia was most prevalent in those aged 19–39 years, but the rates of awareness, treatment, and control among all patients with dyslipidemia in this age group were significantly lower than those in other age groups. The predictors of optimal control were age ≥ 40 years [range 40–49 years: adjusted odds ratio (aOR) 3.73, 95% confidence interval (CI) 1.43–9.72; 50–59 years: aOR 6.25, 95% CI 2.50–15.65; 60–69 years: aOR 6.96, 95% CI 2.77–17.44; 70–79 years: aOR 9.21, 95% CI 3.58–23.74; and ≥ 80 years: aOR 4.43, 95% CI 1.60–12.27]; urban living (aOR 1.44, 95% CI 1.15–1.80); higher body mass index (aOR 1.27, 95% CI 1.13–1.42); lower glycated hemoglobin levels (aOR 0.71, 95% CI 0.67–0.76); hypertension (aOR 1.53, 95% CI 1.22–1.92); poorer self-rated health status (aOR 0.72, 95% CI 0.62–0.84); and receiving regular health check-ups (aOR 1.58, 95% CI 1.25–2.00).

**Conclusions:**

Most patients with DM were diagnosed with dyslipidemia, but many were unaware of or untreated for their condition. Therefore, their control rate was suboptimal. Thus, by understanding factors influencing optimal control of dyslipidemia, physicians should make more effort to encourage patients to undergo treatment and thus, adequately control their dyslipidemia.

## Background

Cardiovascular disease (CVD) continues to be the leading cause of morbidity and mortality in patients with diabetes mellitus (DM) worldwide [1–3]. It is well known that patients with DM have a substantially higher risk of developing CVD than the general population [4, 5]. In addition, dyslipidemia, which is a major risk factor for CVD [6, 7], is notably prevalent in patients with DM [8]. Considering that the optimal control of dyslipidemia is fundamental for the primary prevention of CVD [9, 10], patients with DM need a more effective management of dyslipidemia than those without DM. The 2018 American College of Cardiology/American Heart Association and the 2019 European Society of Cardiology/European Atherosclerosis Society guidelines for the management of dyslipidemia have stratified patients with DM as having a higher risk for developing atherosclerotic CVDs and recommend stricter low-density lipid cholesterol (LDL-C) control [11, 12]. The 2018 Korean Society of Lipid and Atherosclerosis (KSoLA) dyslipidemia management guidelines, based on the National Cholesterol Education Program-Adult Treatment Panel III, has classified patients with DM without CVD as a high-risk group and suggested that an LDL-C goal of < 100 mg/dL should be maintained [13, 14]. Despite these recommendations, previous studies have reported suboptimal achievement of LDL-C goal among patients with DM with dyslipidemia [15–17]. Furthermore, in contrast to previous studies that investigated the prevalence, awareness, treatment, and control rates of dyslipidemia [18–21], relatively few studies have evaluated the specific rates of dyslipidemia in patients with DM, especially in Korea. Therefore, this study aimed to examine the prevalence, awareness, treatment, and control of dyslipidemia among patients with DM using data from the Korea National Health and Nutrition Survey (KNHANES), which is a nationally representative population-based survey. In addition, this study attempted to identify factors associated with the optimal control of dyslipidemia to provide an essential perspective for the primary prevention of CVD among patients with DM.

## Methods

### Data source and study population

**Fig. 1:**
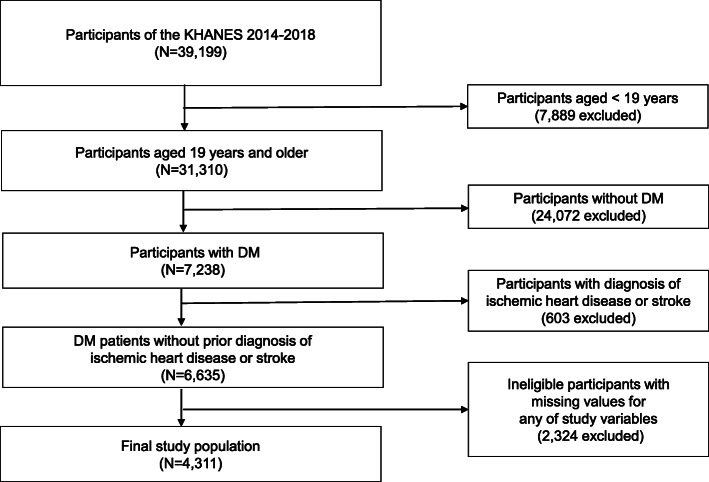
Flow diagram of selection of study population. KNHANES, Korea National Health and Nutrition Examination Survey; DM, diabetes mellitus

The data used in this study were retrieved from the KNHANES from 2014 to 2018. The KNHANES is a national surveillance program that has been conducted annually by the Korea Centers for Disease Control and Prevention (KCDC) since 1998 to evaluate the health and nutritional status of the general population of Korea. The selection process of participants for KNHANES involves complex, stratified, multistage, and cluster sampling to assemble unbiased nationally representative data [22, 23]. This surveillance program consists of surveys using a health interview, health examination, and nutritional status. The details of the KNHANES, including its representatives, have been mentioned in previous studies [22, 24]. Among the total participants (*n* = 39,199) of the KNHANES from 2014 to 2018, patients with DM aged ≥19 years were selected (*n* = 7238). Patients with DM were defined as those with a fasting blood sugar (FBS) ≥126 mg/dL or glycated hemoglobin (HbA1c) ≥6.5%, currently using oral hypoglycemic agents or insulin, or previously diagnosed with DM by a doctor. Moreover, since the purpose of this study was to evaluate the prevalence and management of dyslipidemia among patients with DM for the primary prevention of CVD, participants who responded positively to the survey questions regarding prior diagnosis of ischemic heart disease (myocardial infarction or angina pectoris) or stroke by a doctor were excluded (*n* = 603). In addition, participants with missing values for any of the study variables, including outcome variables and covariates, were also excluded (*n* = 2324). Therefore, a total of 4311 patients were selected as the final study population (Fig. 1).

### Definitions of study variables

The prevalence of dyslipidemia was defined as the proportion of participants with LDL-C levels higher than the target level (LDL-C ≥ 100 mg/dL) recommended by the KSoLA 2018 guidelines for patients with DM, currently using lipid-lowering agents, or previously diagnosed with dyslipidemia by a doctor. Awareness of dyslipidemia was defined as those who indicated a positive response to the survey regarding prior diagnosis of dyslipidemia by a doctor among those with dyslipidemia. Treatment of dyslipidemia was defined as a positive response to the current use of lipid-lowering agents among patients with dyslipidemia. Control of dyslipidemia was defined as the achievement of LDL-C goal of < 100 mg/dL among those with dyslipidemia. The control rate among patients treated for dyslipidemia was also measured. Hypertension was defined as systolic blood pressure ≥ 140 mmHg or diastolic blood pressure ≥ 90 mmHg, self-reported current use of antihypertensive medication, or self-reported prior diagnosis of hypertension by a doctor. Obesity status was defined using body mass index (BMI) in accordance with the obesity standards in Korea (< 18.5 kg/m^2^ for underweight, 18.5–22.9 kg/ m^2^ for ideal weight, 23–24.9 kg/m^2^ for overweight, and ≥ 25.0 kg/m^2^ for obese) [25]. Family history of CVD was defined as a positive response on the survey to questions regarding their parents or siblings being diagnosed with ischemic heart disease (myocardial infarction or angina pectoris) or stroke. In terms of smoking status, all participants were classified as either “non-smokers” (never and past smokers) or “current smokers.” Drinking status was categorized as either “risky drinker” or “non-risky drinker.” Risky drinkers were defined as participants who consumed alcohol ≥2 times/week, with an average of ≥5 and ≥ 7 drinks/occasion for women and men, respectively. Non-risky drinkers were defined as those who consumed lesser alcohol than risky drinkers. The participants’ physical activity status was classified into two groups according to the Physical Activity Guidelines for Americans, 2nd edition [26]. The guideline recommends that adults need to perform moderate-intensity aerobic physical activity for at least 150 min/week or vigorous-intensity aerobic physical activity for at least 75 min/week or an equivalent mix of moderate and vigorous-intensity activities [26] Participants who satisfied any of these criteria were categorized into the “sufficient physical activity” group. The participants’ responses to the survey question regarding having undergone a health check-up within the last 2 years was used to assess whether they were receiving regular health check-ups.

### Anthropometric and biochemical measurements

Blood samples from every participant were obtained after at least 8 h of fasting [12 h for triglyceride (TG)]. The obtained blood samples were processed, immediately refrigerated, and sent to the central laboratory for cold storage. All received samples were analyzed within 24 h. Total cholesterol (TC), high-density lipoprotein cholesterol (HDL-C), TGs, and FBS levels were assessed using a Hitachi Autonomic Analyzer 7600–210 (Hitachi, Japan), whereas HbA1c level was determined using a Tosoh G8 (Tosoh, Japan). The LDL-C level was directly measured if an analyzer yielded a TG level of ≥200 mg/dL, whereas it was indirectly measured using the Friedewald formula (LDL-C = TC - HDL-C + TG/5) if the TG level was < 200 mg/dL [20, 27]. The Friedewald formula was also used to calculate the LDL-C levels of participants with TC, TG, and HDL-C values, but not LDL-C values. BMI was calculated as an individual’s weight in kilograms divided by height in meters squared. Blood pressure (BP) was measured three times using a mercury sphygmomanometer (Baumanometer; WA Baum Co Inc., Coptague, NY, USA) on the participants’ right arm while they were in a seated position. A resting period of at least 5 min was required before every measurement was taken. The average of the second and third BP readings was recorded as the final BP [20].

### Predictors of optimal control of dyslipidemia

The potential factors that could influence the optimal control of dyslipidemia among patients with DM were categorized into three factors: socio-demographic (age, sex, marital status, educational status, employment status, family income, residential area, and type of medical insurance), health status (BMI, HbA1c, BP, level of self-rated health, prevalence of hypertension, and family history of CVD), and health behavior (smoking status, drinking status, level of physical exercise, and health check-ups within 2 years). The definitions of hypertension and calculation of BMI have been described previously. Other factors were determined based on the self-reported data from the KNHANES health interview survey.

### Statistical analysis

Sampling weights utilized in the sample design of KNHANES were applied to all statistical analyses in this study to represent the entire adult population of Korea without bias [22]. Descriptive analyses were performed to assess the baseline characteristics of the participants and the outcome variables. Continuous variables were expressed as means and standard deviations, whereas categorical variables were expressed as percentages and standard errors. After measuring the prevalence, awareness, treatment, and control rates of dyslipidemia among patients with DM, additional subgroup analyses were performed to compare the rates based on sex and age group. Differences in the rates between subgroups were compared using the chi-square test. To investigate the factors influencing the optimal control of dyslipidemia in patients with DM, initially, univariate logistic regression analyses between patients with controlled and uncontrolled dyslipidemia for each specific factor mentioned above were performed. Subsequently, adjusted multivariate logistic regression analyses using factors with *P* value < 0.1 in the univariate analyses were conducted. All statistical analyses were performed using STATA version 14.1 (Stata Corp., College Station, TX, USA). A two-sided *P* value < 0.05 was considered significant.

## Results

### Baseline characteristics

Of the total 4311 patients with DM without a history of CVD, 49.0% were men and 51.0% were women. The mean age of all participants was 56.4 ± 15.8 years with 68.9% being ≥50 years. A total of 69.9% of all participants were married, whereas 30.1% were single/divorced/separated/widowed. In terms of educational status, 40.5% had a middle school or lower education, 29.3% had a high school education, and 30.3% had a college or higher degree. A total of 55.7% of all participants were employed. In terms of socioeconomic status, 53.5% belonged to the lower middle or low-income class and 60.3% resided in urban areas. A total of 92.7% of all participants were beneficiaries of Medicare. In terms of obesity status, 68.2% were either overweight or obese. In terms of hyperglycemic markers, 47.8% had FBS ≥130 mg/dL and 44.2% had HbA1c ≥8.0%. In contrast, only 20.1% had HbA1c < 6.5%. A total of 32.4% of all participants had LDL-C levels < 100 mg/dL. Also, a total of 80.3% of all participants had BP < 140/90 mmHg. A total of 28.0, 51.8 and 20.3% of all participants rated their health status as poor or very poor, fair, and good or excellent, respectively. A total of 50.6% of all patients had hypertension and 17.9% had a family history of CVD. In terms of health behaviors, 79.0% were non-smokers and 85.9% were non-risky drinkers. A total of 57.9% of all patients had insufficient physical activity levels, whereas 64.8% had undergone health check-ups within 2 years (Table 1).

**Table 1:** Baseline characteristics (*n* = 4311)

Characteristics	Total subjects % (SE) or mean ± SD
Socio-demographic factors	
Sex	
Male	49.0 (0.9)
Female	51.0 (0.9)
Age (years)	56.4 ± 15.8
19–39	15.5 (0.8)
40–49	15.8 (0.8)
50–59	23.8 (0.8)
60–69	21.1 (0.7)
70–79	17.0 (0.6)
≥ 80	7.0 (0.4)
Marital status	
Married	69.9 (1.0)
Single/divorced/seperated/widowed	30.1 (1.0)
Educational status	
Middle school or lower	40.5 (1.0)
High school	29.3 (0.9)
College or higher	30.3% (1.0)
Employment status	
Yes	55.7 (0.9)
No	44.3 (0.9)
Family Income	
Low	27.6 (0.9)
Lower middle	25.9 (0.9)
Upper middle	23.5 (0.8)
High	23.0 (0.9)
Residential area	
Urban	60.3 (1.2)
Rural	39.7 (1.2)
Medical insurance	
Medicare	92.7 (0.6)
Medical aid	7.3 (0.6)
Health-related factors	
BMI (kg/m^2^)	
8.5	2.4 (0.3)
18.5–22.9	29.5 (0.8)
23–24.9	22.2 (0.7)
≥ 25	46.0 (0.9)
FBS (mg/dL)	
< 130	31.3 (0.8)
130–179	29.9 (0.9)
≥ 180	38.8 (1.0)
HbA1c (%)	
< 6.5	20.1 (0.8)
6.5–6.9	19.0 (0.7)
7.0–7.4	11.1 (0.6)
7.5–7.9	5.6 (0.4)
≥ 8.0	44.2 (1.0)
LDL-C (mg/dL)	
< 100	32.4 (0.9)
100–129	19.8 (0.7)
130–159	12.7 (0.6)
≥ 160	35.1 (1.0)
Blood pressure (mmHg)	
< 120/80	38.7 (1.0)
120/80–139/89	41.6 (0.9)
≥ 140/90	19.7 (0.8)
Self-rated health	
Very poor/poor	28.0 (0.8)
Fair	51.8 (0.9)
Good/Excellent	20.3 (0.7)
Hypertension	
Yes	50.6 (0.9)
No	49.4 (0.9)
Dyslipidemia	
Yes	83.3 (0.7)
No	16.7 (0.7)
Family history of CVD	
Yes	17.9 (0.7)
No	82.1 (0.7)
Health behavior factors	
Smoking status	
Never/past smoker	79.0 (0.8)
Current smoker	21.0 (0.8)
Drinking status	
Non-risky drinking	85.9 (0.7)
Risky drinking	14.1 (0.7)
Physical activity	
Sufficient	42.1 (0.9)
Insufficient	57.9 (0.9)
Health check-up within 2 years	
Yes	64.8 (0.9)
No	35.2 (0.9)

All data were weighted to the Korean standard population

Abbreviations: *SE* Standard error, *SD* Standard deviation, *BMI* Body mass index, *FBS* Fasting blood sugar; *HbA1c* Glycated hemoglobin, *LDL-C* Low density lipoprotein cholesterol, *SBP* Systolic blood pressure, *DBP* Diastolic blood

### Prevalence, awareness, treatment, and control rates of dyslipidemia among patients with DM

The prevalence of dyslipidemia among all patients with DM was 83.3%, whereas the awareness and treatment rates of dyslipidemia were 36.5 and 26.9%, respectively. The control rate of all patients with dyslipidemia was 18.8% and it was 61.1% among those taking lipid-lowering agents. In the subgroup analysis, in terms of sex, women had significantly higher prevalence (88.3% vs. 78.1%) and awareness rates (38.3% vs. 34.3%) of dyslipidemia than those in men. The differences of treatment and control rates (both control among all patients with dyslipidemia and control among treated patients) between men and women were all statistically insignificant. In the subgroup analysis in terms of age group, dyslipidemia was most prevalent (88.5%) in patients aged < 40 years. The prevalence in other age groups was similar, ranging from 79.9 to 83.4%. Despite having the highest prevalence, participants aged < 40 years had the lowest awareness (4.7%), treatment (3.1%), and control rates among all patients with dyslipidemia (2.2%) compared to those in the other age groups. In contrast, older participants (aged 40–79 years) tended to have more improved rates. However, all these rates were significantly decreased in patients aged ≥80 years. Among patients who were treated for dyslipidemia, those aged < 40 years had the highest control rate (73.7%), while other age groups had generally similar rates, ranging from 58.5 to 62.7% (Table 2).

**Table 2:** The rates of prevalence, awareness, treatment, and control of dyslipidemia among patients with diabetes mellitus by age and sex

Variables	All % (SE)	By sex			By age						
		Men % (SE)	Women % (SE)	*P* value	19–39 years % (SE)	40–49 years % (SE)	50–59 years % (SE)	60–69 years % (SE)	70–79 years % (SE)	≥80 years % (SE)	*P* value
Prevalence^a^	83.3 (0.7)	78.1 (1.2)	88.3 (0.8)	0.000	88.5 (1.7)	82.6 (2.0)	83.3 (1.5)	82.6 (1.4)	79.9 (1.4)	83.4 (2.3)	0.019
Awareness^b^	36.5 (1.0)	34.3 (1.4)	38.3 (1.3)	0.038	4.7 (1.2)	28.6 (2.7)	48.9 (2.1)	50.2 (1.9)	49.1 (2.1)	22.9 (2.7)	0.000
Treatment^b^	26.9 (0.9)	25.8 (1.3)	27.9 (1.1)	0.232	3.1 (1.1)	19.1 (2.4)	31.0 (2.0)	38.8 (1.8)	40.6 (2.1)	19.7 (2.6)	0.000
Control^b^	18.8 (0.8)	18.9 (1.2)	18.7 (1.0)	0.873	2.2 (1.0)	13.5 (1.9)	22.6 (1.8)	25.5 (1.8)	29.4 (2.0)	12.3 (2.0)	0.000
Control among treated^c^	61.1 (1.8)	63.6 (2.8)	59.2 (2.2)	0.207	73.7 (12.9)	60.2 (5.9)	62.6 (3.7)	58.5 (3.1)	62.7 (3.1)	58.8 (6.6)	0.000

All data were weighted to the Korean standard population

^a^*n* = 4311

^b^*n* = 3565

^c^*n* = 1090

*P* values were calculated by chi-square test

Abbreviation: *SE* Standard error

### Factors associated with optimal control of dyslipidemia among patients with DM

The results of the univariate and multivariate logistic regression analyses on the factors influencing optimal control (LDL-C level < 100 mg/dL) of dyslipidemia among patients with DM are presented in Table 3. In the univariate analysis, age ≥ 40 years was the most significant factor associated with optimal dyslipidemia control. The odds ratios (ORs) of patients in older age groups were significantly higher than those of the baseline group of patients aged ≤40 years. The overall trend of OR in relation to age was consistent with that seen in the dyslipidemia control rates. The OR increased with age, peaking at 70–79 years, and markedly decreased thereafter [OR 6.79, 95% confidence interval (CI) 2.64–17.49 for 40–49 years; OR 12.71, 95% CI 5.13–31.47 for 50–59 years; OR 14.84, 95% CI 6.03–36.54 for 60–69 years; OR 18.06, 95% CI 7.29–44.75 for 70–79 years; OR 6.09, 95% CI 2.33–15.95 for ≥80 years]. Married status (OR 1.49, 95% CI 1.20–1.86), urban living (OR 1.53, 95% CI 1.24–1.88), higher BMI (OR 1.38, 95% CI 1.25–1.52), hypertension (OR 2.52, 95% CI 2.07–3.06), a family history of CVD (OR 1.29, 95% CI 1.02–1.64), and having undergone a health screening within 2 years (OR 2.03, 95% CI 1.63–2.52) were also significantly associated with optimal dyslipidemia control. Meanwhile, higher education (OR 0.72, 95% CI 0.64–0.81), better self-rated health status (OR 0.67, 95% CI 0.59–0.77), and current smoking status (OR 0.70, 95% CI 0.53–0.91) were significantly correlated with suboptimal control of dyslipidemia. In the multivariate analysis, an age ≥ 40 years remained the most significant determinant for the optimal control of dyslipidemia. The trend in the adjusted ORs of the older age group as compared to those of the 19–39 years age group was also consistent with the trend seen in the univariate analysis [adjusted OR (aOR) 3.73, 95% CI 1.43–9.72 for 40–49 years; aOR 6.25, 95% CI 2.50–15.65 for 50–59 years; aOR 6.96, 95% CI 2.77–17.44 for 60–69 years; aOR 9.21, 95% CI 3.58–23.74 for 70–79 years; and aOR 4.43, 95% CI 1.60–12.27 for ≥80 years]. Urban living (aOR 1.44, 95% CI 1.15–1.80), higher BMI (aOR 1.27, 95% CI 1.13–1.42), hypertension (aOR 1.53, 95% CI 1.22–1.92), and having undergone a health screening within 2 years (aOR 1.58, 95% CI 1.25–2.00) were also identified as independent predictors of the optimal control of dyslipidemia. On the other hand, higher HbA1c (aOR 0.71, 95% CI 0.67–0.76) and better self-rated health status (aOR 0.72, 95% CI 0.62–0.84) were independently associated with suboptimal control of dyslipidemia. The other factors with notable correlations in the univariate analysis were not significantly corelated in the multivariate analysis.

**Table 3:** Factors associated with optimal control (LDL-C < 100 mg/dL) of dyslipidemia among patients with diabetes mellitus (*n* = 3565)

Factors	Univariate logistic regression analysis		Multivariate logistic regression analysis	
	Crude OR (95% CI)	*P* value	Adjusted OR^a^(95% CI)	*P* value
Socio-demographic factors				
Age				
< 40	1 (reference)		1 (reference)	
40–49	6.79 (2.64–17.49)	0.000	3.73 (1.43–9.72)	0.007
50–59	12.71 (5.13–31.47)	0.000	6.25 (2.50–15.65)	0.000
60–69	14.84 (6.03–36.54)	0.000	6.96 (2.77–17.44)	0.000
70–79	18.06 (7.29–44.75)	0.000	9.21 (3.58–23.74)	0.000
≥ 80	6.09 (2.33–15.95)	0.000	4.43 (1.60–12.27)	0.004
Female	0.98 (0.81–1.19)	0.873		
Married	1.49 (1.20–1.86)	0.000	1.22 (0.95–1.57)	0.114
Employed	0.98 (0.81–1.19)	0.850		
Higher education	0.72 (0.64–0.81)	0.000	1.04 (0.90–1.20)	0.588
Family income	0.98 (0.91–1.06)	0.667		
Urban living	1.53 (1.24–1.88)	0.000	1.44 (1.15–1.80)	0.002
Medicare	1.27 (0.88–1.85)	0.202		
Health-related factors				
Higher BMI (kg/m^2^)	1.38 (1.25–1.52)	0.000	1.27 (1.13–1.42)	0.000
Higher HbA1c (%)	0.65 (0.62–0.69)	0.000	0.71 (0.67–0.76)	0.000
Higher blood pressure (mmHg)	0.95 (0.84–1.08)	0.418		
Hypertension	2.52 (2.07–3.06)	0.000	1.53 (1.22–1.92)	0.000
Positive family history of CVD	1.29 (1.02–1.64)	0.031	1.01 (0.79–1.30)	0.940
Better self-rated health	0.67 (0.59–0.77)	0.000	0.72 (0.62–0.84)	0.000
Health behavior factors				
Current smoker	0.70 (0.53–0.91)	0.008	0.79 (0.59–1.06)	0.118
Risky drinking	0.85 (0.61–1.19)	0.335		
Sufficient physical activity	0.93 (0.76–1.14)	0.482		
Health check-up within 2 years	2.03 (1.63–2.52)	0.000	1.58 (1.25–2.00)	0.000

All data were weighted to the Korean standard population

^a^Adjusted for age, marital status, educational status, residential area, body mass index, HbA1c, prevalence of hypertension, family history of CVD, level of self-related health, smoking status, and health check-up within 2 years

Abbreviations: *LDL-C* Low density lipoprotein cholesterol, *OR* Odds ratio, *CI* Confidence interval; *HbA1c* Glycated hemoglobin, *CVD* Cardiovascular disease

## Discussion

In this nationwide cross-sectional study, dyslipidemia was prevalent in majority (83.3%) of the patients with DM. These numbers were mostly consistent with previous nationwide cross-sectional studies that were performed in Thailand and The United States, with the prevalence of dyslipidemia among patients with DM in those studies being 88.9 and 74.9%, respectively [28, 29]. The slight difference in the prevalence of dyslipidemia in those studies compared to the current study may be due to differences in the definition of prevalence. The study based in Thailand included abnormal TC, TG, HDL-C and LDL-C levels for defining dyslipidemia, while the study based in The United States defined it solely using LDL-C levels and excluded patients maintaining optimal LDL-C levels through medication or lifestyle modification. Thus, considering the definition of dyslipidemia in this study (LDL-C level ≥ 100 mg/dL, a prior diagnosis of dyslipidemia by a doctor, or currently taking lipid-lowering agents), it can be said that the overall prevalence of dyslipidemia in patients with DM would be similar if the same definition was applied to each study. Furthermore, consistent with the findings of previous studies [28, 29], the findings of this study demonstrated that dyslipidemia was more common in women than in men with DM.

Despite the high prevalence of dyslipidemia among patients with DM, the results of the present study indicated that only 36.5% of patients were aware of their disease and that only 26.9% were receiving treatment. According to a recent European longitudinal study, individuals who were unaware of their hypercholesterolemia demonstrated a significantly higher arterial augmentation index than those who were aware of their condition [30]. Thus, it is crucial to educate patients with DM regarding their increased risk of developing dyslipidemia and its associated CVD complications. In terms of control of dyslipidemia, the LDL-C goal (< 100 mg/dL) attainment for patients with DM with dyslipidemia was significantly suboptimal in this study, with an overall control rate of only 18.8%. This value was lower than that reported in a previous study in Korea conducted with the National Health Insurance Service-National Health Screening Cohort (NHIS-HEALS), which reported that 32.6% of patients with DM attained an LDL-C goal of < 100 mg/dL [31]. However, the above mentioned study included all patients with DM regardless of the prevalence of dyslipidemia, whereas the current study focused on control rates of dyslipidemia among patients with DM. In fact, the LDL-C goal attainment rate of all patients with DM regardless of dyslipidemia in the present study was similar to that in the NHIS-HEALS study (32.4%). Therefore, it can be concluded that the rates of uncontrolled dyslipidemia among patients with DM are quite high. Hence, physicians should put forth efforts to motivate patients with DM to optimally control their dyslipidemia in order to prevent development of CVD. In addition, the present study identified that nearly 40% of patients with DM who were taking lipid-lowering agents also failed to achieve an LDL-C goal of < 100 mg/dL. Thus, it is imperative to administer higher-intensity lipid-lowering agents and encourage patients to adhere to their medications.

Interestingly, the subgroup analysis in the current study showed that dyslipidemia was most prevalent in patients with DM aged < 40 years, with nearly 90% of participants in this age group having the condition. In contrast, previous studies conducted with the KNHANES reported that the prevalence of dyslipidemia in all patients aged < 40 years was approximately 15–20% [20, 32], suggesting that patients with DM tend to develop dyslipidemia at an earlier age than the general population. Relatively few studies have analyzed the prevalence of dyslipidemia among patients with DM in terms of age group. A similar trend was also found in the study based in Thailand, with the prevalence of dyslipidemia in patients with DM aged < 50 years being higher than that in those aged ≥50 years (91.1% vs. 88.5%, respectively) [28]. Despite this, patients with DM aged 19–39 years had the lowest awareness, treatment, and control rates. In addition, the rates showed considerable differences in contrast with the patients in other age groups, especially compared to those aged 50–79 years. In terms of the degree of control among those treated for dyslipidemia, rates were actually found to be the highest in the patients with DM aged 19–39 years. However, considering that awareness, treatment, and control rates among patients with DM with dyslipidemia were remarkably lower in this age group than in other age groups, it can be assumed that the status of dyslipidemia control in this age group is generally very poor, except for a few health-conscious individuals who are taking lipid-lowering agents. A previous large-scale retrospective cohort study in Korea showed that increased cholesterol levels were significantly associated with higher CVD risk in young adults aged 20–39 years [33]. The risk of CVD is even higher in young patients with DM and increased cholesterol levels. Thus, more attention should be given to this younger age group with DM in whom dyslipidemia is more prevalent, and its management is inadequate.

With regard to the factors associated with the optimal control of dyslipidemia among patients with DM, the present study identified that age ≥ 40 years, urban living, higher BMI, lower HbA1c, hypertension, poorer self-rated health, and regular health screening were independent predictors for the attainment of the LDL-C goal of < 100 mg/dL, with age being the most prominent factor. In terms of age, the aORs for the optimal control of dyslipidemia significantly increased with age until 79 years. This trend could possibly be interpreted by the “health belief model” [34, 35], which explains that those who perceive that they are more ill will engage in healthier behaviors to reduce their risk of developing an illness and vice versa. Older age is a well-known risk factor for CVD [11–13], and older patients with DM are likely to have more comorbidities than younger patients. Thus, patients aged ≥40 years are probably more health-conscious than those aged 19–39 years, resulting in better dyslipidemia control. Older age was also an independent factor for non-attainment of LDL-C goal in patients with acute coronary syndrome [36]. The decrease in aOR for optimal dyslipidemia control in patients aged ≥80 years may be due to their lack of motivation for strict lipid control. Compared to the younger age groups, patients aged ≥80 years may think that they are nearing the end of their lifespan. The fact that the awareness and treatment rates of patients in this age group were significantly lower than those in patients aged 50–70 years, may strengthen this assumption. The health belief model could also be used to explain the other positive predictors of optimal dyslipidemia control, such as higher BMI, hypertension, poorer self-rated health, and health screening within 2 years. Patients with these factors are likely to be more concerned with their general health status due to their underlying conditions (e.g., obesity and hypertension) or higher perceived susceptibility to developing specific health problems (e.g., CVD), which consequently affected their behaviors for better control of dyslipidemia. Meanwhile, the “healthy adherer effect” [37] may explain how lower HbA1c levels may predict better control of dyslipidemia. Patients with well-controlled DM are more likely to exhibit other healthy behaviors, leading to better control of dyslipidemia. Finally, rural residents are more likely to face barriers in accessing healthcare due to limited medical centers, fewer physicians, and transportation difficulties than patients living in urban areas [38, 39], which would lead to poorer control of dyslipidemia. Thus, clinicians should pay extra attention when managing dyslipidemia in patients with DM aged < 40 years, residing in rural areas, not undergoing regular health screening, with poor hyperglycemic control, no hypertension, lower BMI, or better self-rated health since they are at higher risk of poor dyslipidemia control.

### Study strengths and limitations

The strength of this study was that it used nationally representative KNHANES data to objectively estimate the recent trends in the prevalence and management of dyslipidemia among adults with DM in Korea. In addition, various factors that could potentially influence the control of dyslipidemia were analyzed to provide a meaningful perspective for better LDL-C control in patients with DM. However, this study has several limitations. First, due to its cross-sectional nature, longitudinal follow-up of participants could not be conducted. Second, majority of the data used in this study were based on a self-administered questionnaire. Hence, the possibility of recall bias and unintentional errors cannot be ruled out. Third, the control rate of dyslipidemia among patients with DM could have been partially overestimated. The KSoLA guidelines recommend that the LDL-C goal for patients with DM may be lowered for patients who have target organ damage or major CVD risk factor. However, participants with target organ damage could not be distinguished in this study due to lack of information in the KNHANES data. However, the KSoLA guidelines do not mention the specific LDL-C goal for patients with target organ damage or major CVD risk factors. Hence, patients with DM with dyslipidemia in Korea are generally managed with an LDL-C goal < 100 mg/dL. Other studies based in Korea that examined LDL-C goal attainment in patients with dyslipidemia also used LDL-C goal < 100 mg/dL when assessing control of dyslipidemia among patients with DM without CVD [31, 40]. In addition, the control rate of patients with DM in this study was already poor despite potential overestimation. Thus, it can be considered that the findings of this study raise potential concerns about poor dyslipidemia control among patients with DM in Korea. Lastly, due to the lack of information in the KNHANES data, the interaction between the patient and physician, which could also be an important factor in the control of dyslipidemia, could not be evaluated.

## Conclusions

In this nationwide cross-sectional study, majority of patients with DM were diagnosed with dyslipidemia, but a large proportion of them were not aware of or remained untreated for their condition. Therefore, their control of dyslipidemia was generally suboptimal. Age ≥ 40 years, urban living, higher BMI, lower HbA1c, hypertension, poorer self-rated health, and undergoing regular health screening were independent predictors of optimal dyslipidemia control. Thus, more efforts should be made to encourage patients with DM to undergo treatment and thereby adequately controlling their dyslipidemia, especially when managing patients aged < 40 years, with poor hyperglycemic control, no hypertension, lower BMI, better self-rated health, residing in rural areas, or not receiving regular health screening for primary prevention of CVD.

## Data Availability

The data used in this study was obtained from the KNHANES (Korea National Health and Nutrition Examination Survey. These data can be download from the following website: https://knhanes.cdc.go.kr/knhanes/eng/index.do; after submission and evaluation of an appropriate research protocol.

## Abbreviations

aOR: Adjusted odds ratio
BMI: Body mass index
BP: Blood pressure
CI: Confidence interval
CVD: Cardiovascular disease
DM: Diabetes mellitus
HDL-C: High-density lipoprotein cholesterol
FBS: Fasting blood sugar
IRB: Institutional review board
KCDC: Korea Centers for Disease Control and Prevention
KNHANES: Korea National Health and Nutrition Survey
KSoLA: Korean Society of Lipid and Atherosclerosis
LDL-C: Low-density lipid cholesterol
NHIS-HEALS: National Health Insurance Service-National Health Screening Cohort
OR: Odds ratio
TC: Total cholesterol
TG: Triglyceride
